# Age-Adjusted Cut-Off Values for Glial Fibrillary Acidic Protein and Ubiquitin Carboxy-Terminal Hydrolase L1 Improve the Diagnostic Accuracy of the Abbott Mild Traumatic Brain Injury Assay

**DOI:** 10.3390/diagnostics15091093

**Published:** 2025-04-25

**Authors:** Ivana Lapić, Dunja Rogić, Matea Bingula, Lea Miklić, Ivan Gornik

**Affiliations:** 1 Department of Laboratory Diagnostics, University Hospital Centre Zagreb, 10000 Zagreb, Croatia; predstojnik.lab@kbc-zagreb.hr; 2 Faculty of Pharmacy and Biochemistry, University of Zagreb, 10000 Zagreb, Croatia; 3 Department of Emergency Medicine, University Hospital Centre Zagreb, 10000 Zagreb, Croatia; matea.bingula1@kbc-zagreb.hr (M.B.); lea.miklic@kbc-zagreb.hr (L.M.); ivan.gornik@gmail.com (I.G.); 4 School of Medicine, University of Zagreb, 10000 Zagreb, Croatia

**Keywords:** mild traumatic brain injury, emergency department, biomarkers, computed tomography

## Abstract

**Objectives**: To establish age-adjusted cut-off values for glial fibrillary acidic protein (GFAP) and ubiquitin carboxy-terminal hydrolase L1 (UCH-L1) and assess their impact on the diagnostic performance of the mild traumatic brain injury (mTBI) assay. **Methods**: The study included 175 adult mTBI patients presenting at the emergency department (ED) within 12 h from head trauma in whom head CT scan was performed. GFAP and UCH-L1 were measured using chemiluminescence immunoassays on an Abbott analyzer (Abbott Laboratories, USA). **Results**: Using manufacturer’s defined cut-offs (GFAP < 35 ng/L, UCH-L1 < 400 ng/L), the mTBI assay exhibited diagnostic sensitivity (Se) of 93.1%, specificity (Sp) of 28.8%, negative predictive value (NPV) of 95.5% and a positive predictive value (PPV) of 20.6%. In the subgroup of patients aged under 50, Se and NPV were below 100% (i.e., 75.0% and 92.3%), due to two false negative mTBI results. Age-adjusted cut-offs were defined for three patient groups, ≤49 years, 50–69 years and ≥70 years, and were set to 22.4, 37.0 and 62.3 ng/L for GFAP, and 349.3, 351.6 and 369.0 ng/L for UCH-L1. Using these cut-offs, in all patient groups Se and NPV were 100%, while increased Sp was obtained in patients older than 50 years. **Conclusions**: Diagnostic Se and NPV can be improved by the use of age-adjusted cut-offs. In this way, the triage protocol for mTBI and head CT scan can be refined, further contributing to the optimization of the diagnostic management of mTBI patients at the ED.

## 1. Introduction

Mild traumatic brain injury (mTBI), accounting for 80–90% of all head traumas, is the most common type of brain injury for which patients seek help at the emergency department (ED) [1]. It is defined as a closed head injury caused by a sudden accelerating or decelerating brain movement within the skull, resulting in a transient alteration of mental status, which is presented by temporary neurological symptoms. These include short-term loss of consciousness (<30 min), disorientation or post-traumatic amnesia (<1 day), and are usually accompanied by mild neurological manifestations including headache, dizziness, nausea and/or vomiting [1,2]. In those patients, the Glasgow Coma Scale (GCS), which is the main instrument for classifying the severity of traumatic brain injury, fluctuates between 13 and 15. Despite mild symptomatology, intracranial abnormalities can occur in 10–20% of all patients initially classified as mTBI, hence requiring accurate detection and prompt treatment. Therefore, patients with mTBI invariably undergo head computed tomography (CT) as part of the diagnostic management at the ED. This clearly represents a significant burden for the patient due to possibly unnecessary exposure to radiation and a prolonged stay at the ED, but also for the healthcare system in terms of ED overcrowding, overuse of radiology testing and increased costs.

Blood-based biomarkers that could accurately reflect acute intracranial lesions and reduce the use of head CT scan in patients with mTBI have been widely studied [3]. Numerous studies have shown that the combined determination of glial fibrillary acidic protein (GFAP) and ubiquitin carboxy-terminal hydrolase L1 (UCH-L1) within 12 h from head injury exhibits adequate diagnostic performance in terms of excluding intracranial abnormalities for mTBI adult patients [4,5,6,7,8,9,10,11]. Consequently, this dual test was cleared in 2018 by the United States Food and Drug Administration as being capable of eliminating the need for head CT scans in adult mTBI patients if both results show values below the predefined cut-off. Several studies confirmed its validity for this purpose, uniformly yielding diagnostic sensitivities and negative predictive values above 95% [4,5,6,7,8,9,10,11]. However, in a significant number of patients without acute intracranial lesions, the value of at least one of the two tests was shown to be above the predefined cut-off. This is especially pronounced in the elderly population, due to age-associated neurodegenerative changes and systemic comorbidities [4]. Therefore, it is reasonable to assume that age-adjusted cut-off values could contribute to the more appropriate interpretation of mTBI biomarkers and further optimize the management and workflow of ED patients.

The aim of the present study was to assess the overall diagnostic accuracy of the mTBI assay in a cohort of Croatian patients presenting at the ED with mTBI, as well as to establish age-adjusted cut-off values for GFAP and UCH-L1 and elucidate their impact on the diagnostic performance of the mTBI assay.

## 2. Materials and Methods

### 2.1. Study Setting and Participants

The present study was conducted at the Department of Emergency Medicine, University Hospital Centre Zagreb, Croatia from February to October 2024. The study included adult patients (>18 years) who were admitted to the ED due to an acute head trauma event that happened within 12 h before admission, resulting in mild neurological symptoms such as headache, vertigo, nausea and/or vomiting, short-term loss of consciousness (<30 min), amnesia, confusion and/or disorientation. All consecutive patients with the GCS score between 13 and 15 who underwent a head CT scan as part of the diagnostic management at the ED were included. Exclusion criteria were as follows: head trauma older than 12 h, history of any kind of neurological or psychiatric disorders, previous neurosurgical interventions and/or traumatic brain injuries. Also, patients with severe acute or chronic systemic conditions including gastrointestinal disorders, sepsis, pneumonia, end-stage chronic kidney disease and other non-neurological disorders were not included in the study.

In addition, to evaluate the relevance of establishing age-adjusted cut-off values, the study included a group of age- and sex-matched adult healthy control subjects.

### 2.2. Laboratory Analyses

Measurement of GFAP and UCH-L1 was performed at the emergency laboratory of the Department of Laboratory Diagnostics, University Hospital Centre Zagreb, Croatia. The laboratory is located within the premises of the ED and performs laboratory diagnostics exclusively for outpatients admitted to the ED. The mTBI assay (Abbott Laboratories, Chicago, IL, USA) was applied on the immunoassay module of the Alinity ci (Abbott Laboratories, Chicago, IL, USA) integrated automated analyzer that utilizes the chemiluminescence immunoassay principle. All analyses were performed using the original manufacturer’s application and strictly following the manufacturer’s instructions. The manufacturer’s recommended cut-off values for GFAP and UCH-L1 are 35.0 ng/L and 400.0 ng/L, respectively. The mTBI assay is considered as positive if the result of either of the two parameters falls above the aforementioned cut-off. The assay is regularly monitored with dedicated internal quality control samples at two levels that cover the clinically relevant concentration range. During a 3-month period, the obtained between-day coefficient of variation (CV) for GFAP was 5.2% at the concentration of 25.0 ng/L, and 5.1% at 500.0 ng/L. For UCH-L1, the obtained CVs were 3.2% at 250.0 ng/L concentration, and 4.8% at 2000.0 ng/L.

GFAP and UCH-L1 were measured in blood samples drawn into 3 mL lithium heparin vacutainer Vacuette (Greiner Bio-One, Kremsmünster, Austria) for the purpose of routine laboratory management at the ED. Plasma was obtained by centrifugation 10 min at 2100× *g*, and an aliquot was stored at −20 °C until measurement, which was performed within 30 days. Prior to analysis, plasma samples were thawed in a water bath, mixed using a vortex and recentrifuged to remove any possible fibrin strands or other particles that could interfere with measurement, as well as to obtain sample homogeneity.

### 2.3. Statistical Analysis

Data distribution normality was assessed using the Shapiro–Wilk test and the results are presented as medians and interquartile ranges. The diagnostic accuracy of GFAP and UCH-L1, individually and in combination, in relation to the findings of the head CT scan was determined by the receiver operating characteristics (ROC) analysis. The area under the curve (AUC), diagnostic sensitivities and diagnostic specificities with corresponding 95% confidence intervals (CI) were calculated. The age-adjusted cut-offs for GFAP and UCH-L1 were estimated consecutively. Given the superior diagnostic accuracy characteristics of GFAP over UCH-L1 [5], ROC analysis was firstly conducted for GFAP alone at the manufacturer’s cut-off point. The optimal cut-off was selected according to the Youden index. Using this cut-off, interpretations of both GFAP alone and mTBI were reclassified in terms of positivity and negativity. Afterwards, ROC analysis for UCH-L1 was performed in relation to the latter classification and an optimal cut-off was obtained. Finally, the mTBI interpretation was re-evaluated according to these newly established cut-off values for three age groups (≤50 years, between 50 and 69 years, and ≥70 years) and corresponding diagnostic accuracy data were calculated. Statistical analysis was performed in the MedCalc statistical software, version 19.5.2 (MedCalc, Ostend, Belgium).

## 3. Results

**Figure 1:**
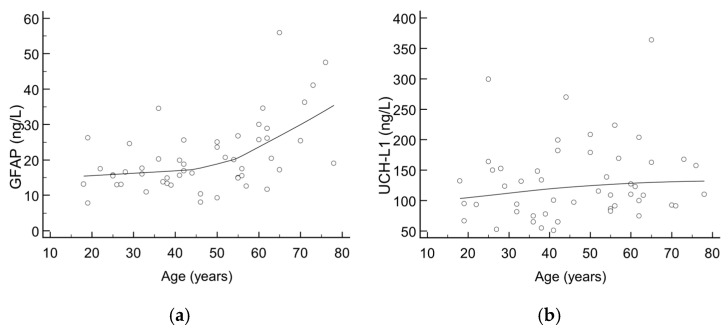
Scatter diagrams showing trend of (**a**) GFAP and (**b**) UCH-L1 values according to age in healthy control subjects.

There were 52 healthy control subjects (median age: 47 years, aged from 18 to 78; 61.5% females) recruited for the purpose of evaluating the trend of GFAP and UCH-L1 according to age. The majority of results for GFAP and all UCH-L1 results were below the cut-offs defined by the manufacturer; however, a trend towards higher values was seen with increasing age, as shown in Figure 1. Upon visual inspection, an inflection point can be seen at an age of around 50 years, being more pronounced for GFAP than UCH-L1.

A total of 175 patients with mTBI admitted to the ED were included in the study, out of which 29 (16.6%) had positive findings on the head CT scan. Detailed patient demographics and clinical characteristics are presented in Table 1.

The results of mTBI assay were positive in 131 (74.9%) patients. Specifically, GFAP was above the predefined cut-off (35.0 ng/L) in 108 patients, UCH-L1 was above 400.0 ng/L in 92 patients, while both assays were elevated in 68 patients. Out of the 29 patients with positive head CT findings, 2 had negative mTBI assay findings. Among the 27 patients with positive CT findings and positive mTBI assay interpretation, 19 had elevated levels of both GFAP and UCH-L1; in 5, only GFAP was above the cut-off, while 3 patients had only UCH-L1 increased. Diagnostic accuracy data of GFAP and UCH-L1, used both individually and in combination, is presented in Table 2.

Study participants were further divided into three subgroups according to age: up to 49 years (*n* = 52), from 50 to 69 (*n* = 44), and over 70 years (*n* = 79). Demographic and clinical characteristics, as well as diagnostic accuracy data for the mTBI assay according to manufacturer’s predefined cut-off values in these three subgroups, are listed in Table 3.

The positivity rate of the mTBI biomarker values was found to be steadily increasing by age. Specifically, in the group up to 49 years, 26/52 (50.0%) had positive interpretation of the mTBI assay, in the group from 50 to 69 years, there were 31/44 (70.5%) patients with positive mTBI assay, while in the group from 70 years and over, in a total of 74/79 (93.7%) patients, a positive mTBI test result was obtained.

Age-adjustment of the cut-off values for GFAP and UCH-L1 increased the diagnostic accuracy in all three subgroups. Specifically, diagnostic sensitivities and negative predictive values of 100% were obtained in all assessed patient subgroups and increases in diagnostic specificities were observed for patients older than 50 years (Table 4). Reclassification of patients according to the newly established cut-off values yielded additional seven negative mTBI findings, but also additional eight positive ones. Importantly, among the eight patients reclassified as having positive mTBI biomarker values were the two patients with positive head CT scans and previously negative mTBI interpretation.

## 4. Discussion

The present study confirms that the mTBI dual test consisting of simultaneous determination of GFAP and UCH-L1 has satisfactory diagnostic sensitivity and negative predictive value. Thus, it can serve as a valuable tool for eliminating the need for head CT scan in patients admitted to the ED with a suspected mTBI. This study additionally points to the fact that adjustment of cut-off values according to age can further improve the diagnostic accuracy of the mTBI test.

Numerous studies have shown that the mTBI test can be safely used as a rule-out test for the exclusion of intracranial abnormalities in adult mTBI patients within 12 h from head trauma [4,5,6,7,8,9,10,11]. However, the diagnostic performance characteristics of the assay slightly differ among studies, depending on the study setting and design, patients inclusion criteria and the type of immunoassay used, as well as assay-specific cut-offs. In the present study, by applying the manufacturer’s defined cut-off values for GFAP and UCH-L1, the mTBI test exhibited a diagnostic sensitivity of 93.1% and a negative predictive value of 95.5%. This performance was slightly worse than data published in previously conducted studies where diagnostic sensitivities were uniformly found to be above 96%, whereas NPVs ranged from 95% to 100% [4,5,6,7,8,9,10,11]. Our result was a direct consequence of the two false negative cases identified among patients younger than 50 years, similar to what was previously reported by Ladang et al. [8], who encountered one false negative case in a young patient. Therefore, when patients were grouped according to age, poorer diagnostic sensitivity was obtained in patients aged below 50, which confirmed the need for age-adjusted cut-off values. On the contrary, in patients older than 50 years, no false negatives were found, and diagnostic sensitivities and NPVs of 100% were achieved. On the other hand, diagnostic specificities throughout published studies did not exceed 40% [4,5,6,7,8,9,10,11], which was also confirmed by our study (28.8% diagnostic specificity). Nevertheless, in our patient series, the use of the mTBI test could have reduced the use of head CT scans by one fourth. Expectedly, a decrease in diagnostic specificities was observed with age, declining to 7.6% in patients older than 70 years, which is a result of a large number of false positive cases, a finding that was also addressed by Ward et al. [12]. Overall, the positivity rate of the mTBI test is known to be high, and it substantially increases with age, due to age-dependent elevations of GFAP and UCH-L1 that are not related to acute head injury events, but rather neurodegenerative alterations which accompany advancing age [4]. Since the incidence of head injury events is the highest in the elderly population, mainly due to the worsening of motor skills and susceptibility for falls, it is reasonable to consider adaptation of diagnostic cut-offs in this group of patients.

The relevance of establishing age-adjusted cut-off values was proven in the present study. The hereby suggested cut-off values for both GFAP and UCH-L1 increase with age and differ from the ones recommended by the manufacturer. Specifically, for GFAP, a lower cut-off compared to that recommended by the manufacturer was suggested only in patients younger than 50 years (i.e., 22.4 ng/L), while with increasing age, the cut-off steadily rose to 37.0 ng/L in patients aged 50 to 69 years and 62.3 ng/L in those older than 70 years. Regarding UCH-L1, a lower cut-off than the one defined by the manufacturer was obtained across the whole patient cohort. These results are in accordance with the study conducted by Ladang et al. [8], who equally suggest lowering of the cut-off for UCH-L1 in patients older than 65 years to 335 ng/L. Similarly, they suggested an increased cut-off for GFAP in the elderly; however, this is almost two-fold higher than the one obtained in our study. These differences can be explained by the heterogeneity of the patient population included head traumas of different severity, which yield variable releases of the studied biomarkers in circulation, as well as different blood sampling time points. Nevertheless, the introduction of age-adjusted cut-offs might eliminate the confounding factor presented by the age-related differences in biomarkers’ concentrations. As indicated by the present study, age-adjusted cut-offs could contribute to improved diagnostic performance of the mTBI test. It is noteworthy that by applying these newly defined cut-offs, diagnostic sensitivities and NPVs of 100% were achieved for all age groups. Furthermore, diagnostic specificities for patients older than 50 years were increased in this way, contributing to an overall improved diagnostic specificity of the mTBI test. However, although age-appropriate cut-off adjustments improved the diagnostic performance of the mTBI test—most importantly by eliminating the false-negative cases—this modification did not increase the rate of ruled-out patients compared to the use of manufacturer’s cut-offs. The rate of false positives remains inherently high, the underlying cause of which remains uncertain. Besides age-associated susceptibility to neurodegeneration, there is also evidence that subclinical neuroaxonal damages not detectable by head CT scan increase the levels of GFAP and UCH-L1 in circulation [13], which would classify them as more sensitive markers of central nervous system injury as compared to CT scans. This might, at least partly, explain the high positivity rate of the mTBI assay in patients with mild head trauma regardless of age.

This study has some limitations. Firstly, it is a single-centre study that included consecutive patients admitted to a single ED in a defined period of time. Secondly, the cohort of patients with mTBI is heterogeneous, of medium size and with a limited number of cases with head CT positive findings; therefore, it is not possible to fully translate the obtained results to another patient population. Hence, in order to prove the relevance of the newly proposed cut-offs, they should be validated in different settings, optimally through a multicentric evaluation, and include a substantially larger population of patients with suspected mTBI. A more detailed evaluation of mTBI biomarkers in relation to serious chronic diseases that may affect their values might also be valuable for a more profound insight into their diagnostic performance. Although a reference method does not exist, it would be reasonable to evaluate the sensitivity and accuracy of this novel fully automated mTBI assay against well-established conventional enzyme-linked immunosorbent assays. Finally, it would be necessary to prospectively evaluate the diagnostic performance of age-adjusted cut-offs.

## 5. Conclusions

In conclusion, the diagnostic validity of the mTBI test has been already proven by numerous studies. However, age-adjusted cut-off values might be considered a more useful approach in results interpretation. This study suggests that the mTBI excellent diagnostic sensitivity and NPV can be further improved by cut-off adjustments according to age. By applying this approach, the triage protocol for mTBI and head CT scans can be further refined, and thus contribute to improving the diagnostic management of mTBI patients presenting at the ED.

## Figures and Tables

**Table 1:** Demographic and clinical characteristics of study participants (*n* = 175).

Age (years)	65 (18–96)
Females, *N* (%)	83 (47.4)
Glasgow Coma Scale score	15 (13–15)
Mechanism of injury, *N* (%)	
- Fall	151 (86.3)
- Car accident	19 (10.9)
- Violence	5 (2.8)
Time from injury to ED admission (hours)	2.0 (0.5–8)
Time from injury to blood collection (hours)	2.5 (1–10)
Positive CT findings, *N* (%)	29 (16.6)
- Subdural hematoma	9/29
- Subarachnoid hemorrhage	7/29
- Nasal bone fractures	6/29
- Paranasal sinus hemorrhage	2/29
- Epidural hematoma	2/29
- Subdural hygroma	1/29
- Intracerebral hematoma	1/29
- Periorbital hematoma	1/29
GFAP (ng/L)	51.7 (23.2–124.1)
UCH-L1 (ng/L)	424.0 (242.0–753.5)
Positive mTBI assay interpretation, *N* (%)	131 (74.9)

GFAP and UCH-L1 are presented as medians and interquartile ranges, while age, Glasgow Coma Scale score and timeframes are presented as medians and ranges. ED—emergency department, CT—computed tomography, GFAP—glial fibrillary acidic protein, UCH-L1—ubiquitin carboxy-terminal hydrolase L1; mTBI—mild traumatic brain injury.

**Table 2:** Diagnostic accuracy data of the individual and combined performance of GFAP and UCH-L1 at manufacturer’s cut-off values for all included study participants (*n* = 175).

	GFAP	UCH-L1	GFAP and UCH-L1 *
AUC (95% CI)	0.626 (0.550–0.698)	0.640 (0.564–0.711)	0.609 (0.533–0.682)
Diagnostic sensitivity (95% CI)	82.8 (64.2–94.2)	75.9 (56.5–89.7)	93.1 (77.2–99.1)
Diagnostic specificity (95% CI)	42.5 (34.3–50.9)	52.1 (43.6–60.4)	28.8 (21.6–36.8)
Negative predictive value (95% CI)	92.5 (84.5–96.6)	91.6 (84.8–95.5)	95.5 (84.3–98.8)
Positive predictive value (95% CI)	22.2 (18.7–26.2)	23.9 (19.4–29.1)	20.6 (18.4–23.0)

* The assay is considered as positive if either of the two parameters is above the cut-off value. GFAP—glial fibrillary acidic protein, UCH-L1—ubiquitin C-terminal hydrolase L1, CI—confidence interval, AUC—area under the curve.

**Table 3:** Diagnostic accuracy data of the mTBI assay at manufacturer’s cut-off values divided per age groups.

Group	≤49 Years (*n* = 52)	50–69 Years (*n* = 44)	≥70 Years (*n* = 79)
Age (years)	36 (18–49)	60 (50–69)	84 (70–96)
Females, *N* (%)	21 (40.4)	15 (34.1)	47 (59.5)
Positive CT, *N* (%)	8 (15.4)	8 (18.2)	13 (16.5)
Positive mTBI assay interpretation, *N* (%)	26 (50.0)	31 (70.5)	74 (93.7)
AUC (95% CI)	0.648 (0.503–0.775)	0.681 (0.523–0.813)	0.538 (0.422–0.651)
Diagnostic sensitivity (95% CI)	75.0 (34.9–96.8)	100 (63.1–100)	100 (75.3–100)
Diagnostic specificity (95% CI)	54.5 (38.8–69.6)	36.1 (20.8–53.8)	7.6 (2.5–16.8)
Negative predictive value (95% CI)	92.3 (77.8–97.6)	100 (75.3–100)	100 (47.8–100)
Positive predictive value (95% CI)	23.1 (15.2–33.4)	25.8 (21.4–30.8)	17.6 (16.6–18.6)

CT—computed tomography, CI—confidence interval, AUC—area under the curve, mTBI—mild traumatic brain injury; Age is reported as medians and interquartile ranges.

**Table 4:** Diagnostic accuracy of the mTBI assay according to age-adjusted cut-off values.

Group (Years Range)	≤49 Years (*n* = 52)	50–69 Years (*n* = 44)	≥70 Years (*n* = 79)	All Patients (*n* = 175)
GFAP cut-off (ng/L)	22.4	37.0	62.3	
UCH-L1 cut-off (ng/L)	349.3	351.6	369.0	
AUC (95% CI)	0.727 (0.586–0.841)	0.708 (0.552–0.835)	0.569 (0.452–0.679)	0.651 (0.575–0.721)
Diagnostic sensitivity (95% CI)	100 (63.1–100)	100 (63.1–100)	100 (75.3–100)	100 (88.1–100)
Diagnostic specificity (95% CI)	45.5 (30.4–61.2)	41.7 (25.5–59.2)	13.6 (6.4–24.3)	30.1 (22.8–38.3)
Negative predictive value (95% CI)	100 (82.4–100)	100 (78.2–100)	100 (66.4–100)	100 (91.8–100)
Positive predictive value (95% CI)	25.0 (20.3–30.4)	27.6 (22.4–33.4)	18.6 (17.2–20.1)	22.1 (20.4–24.0)

GFAP—glial fibrillary acidic protein, UCH-L1—ubiquitin carboxy-terminal hydrolase L1, CI—confidence interval, AUC—area under the curve, mTBI—mild traumatic brain injury.

## Data Availability

The raw data supporting the conclusions of this article will be made available by the authors on request.

## Abbreviations

The following abbreviations are used in this manuscript:

GFAP	Glial fibrillary acidic protein
UCH-L1	Ubiquitin carboxy-terminal hydrolase L1
mTBI	Mild traumatic brain injury
Se	Diagnostic sensitivity
Sp	Diagnostic specificity
ED	Emergency department
CT	Computed tomography
CI	Confidence interval
AUC	Area under the curve
CV	Coefficient of variation
